# Organizational Context Matters: A Research Toolkit for Conducting Standardized Case Studies of Integrated Care Initiatives

**DOI:** 10.5334/ijic.2502

**Published:** 2017-06-27

**Authors:** Jenna M. Evans, Agnes Grudniewicz, Carolyn Steele Gray, Walter P. Wodchis, Peter Carswell, G. Ross Baker

**Affiliations:** 1 Cancer Care Ontario, CA; 2 Institute of Health Policy, Management and Evaluation, University of Toronto, CA; 3 Telfer School of Management, University of Ottawa, CA; 4 Bridgepoint Collaboratory, Lunenfeld-Tanenbaum Research Institute, Sinai Health System, CA; 5 Toronto Rehabilitation Institute, CA; 6 Institute for Clinical Evaluative Sciences, CA; 7 School of Population Health, The University of Auckland, NZ

**Keywords:** case studies, integrated care, integrated delivery system, organizational context, organizational capabilities

## Abstract

**Introduction:**

The variable success of integrated care initiatives has led experts to recommend tailoring design and implementation to the organizational context. Yet, organizational contexts are rarely described, understood, or measured with sufficient depth and breadth in empirical studies or in practice. We thus lack knowledge of when and specifically how organizational contexts matter. To facilitate the accumulation of evidence, we developed a research toolkit for conducting case studies using standardized measures of the (inter-)organizational context for integrating care.

**Theory and Methods:**

We used a multi-method approach to develop the research toolkit: (1) development and validation of the Context and Capabilities for Integrating Care (CCIC) Framework, (2) identification, assessment, and selection of survey instruments, (3) development of document review methods, (4) development of interview guide resources, and (5) pilot testing of the document review guidelines, consolidated survey, and interview guide.

**Results:**

The toolkit provides a framework and measurement tools that examine 18 organizational and inter-organizational factors that affect the implementation and success of integrated care initiatives.

**Discussion and Conclusion:**

The toolkit can be used to characterize and compare organizational contexts across cases and enable comparison of results across studies. This information can enhance our understanding of the influence of organizational contexts, support the transfer of best practices, and help explain why some integrated care initiatives succeed and some fail.

## Introduction

Integrated care initiatives bring together multiple healthcare professionals and organizations to deliver care that is better coordinated, patient-centered, and cost-effective [1]. The variable success of integrated care initiatives has led experts to recommend tailoring their design and implementation to the local context [2,3,4]. However, relatively little is known about what contextual factors are associated with successful integrated care delivery [5,6]. In addition to the broader social, political, economic and cultural environment [7], factors in the organizational context also influence integrated care efforts. However, organizational contexts are rarely described, understood, or measured with sufficient depth and breadth in empirical studies or in practice. The focus of research and evaluation tends to be on measuring the mechanisms of the intervention itself, the extent to which care is integrated, and outcomes. We thus lack knowledge of which organizational factors matter, and when and how they matter.

We use the term “organizational context” broadly to describe the setting in which an integrated care initiative is implemented [8] and to capture all organizational factors that are not a direct part of the initiative [9], such as governance structures, leadership approach, and organizational culture. This definition encompasses both internal organizational factors as well as collective inter-organizational factors needed to leverage and combine resources from multiple organizations in the delivery of integrated care. For example, “leadership approach” can be examined within organizational boundaries (i.e., an organization’s senior management team) as well as at the inter-organizational level (i.e., designated leaders for the integrated care network or partnership).

Comparative case study research provides useful methods for understanding context and explaining why some integrated care initiatives work effectively in some contexts, but not in others [10,11]. Case study research involves the collection of qualitative, and often quantitative, data from various sources to explore the characteristics of one or more organizations, or parts of organizations [12]. To conduct comparative case studies, researchers need standardized tools for collecting comparable data on organizational factors across care providers, settings, and studies. However, we are unaware of any measurement systems that have been developed with the aim of rigorously characterizing and comparing a wide range of organizational capabilities, spanning structural, process and socio-cultural elements, and involving both qualitative and quantitative methods.

In this paper, we describe the development and content of a conceptual framework and research toolkit for conducting standardized, comparable case studies of the organizational context for integrating care. The toolkit provides methods and tools for assessing key organizational and inter-organizational characteristics that affect the implementation and success of integrated care initiatives.

## Methods

A multi-method approach consisting of five stages informed the development of the toolkit: (1) development and validation of the Context and Capabilities for Integrating Care Framework, (2) development of document review guidelines, (3) identification, assessment and selection of survey instruments, (4), development of interview resources, and (5) pilot testing of the document review guidelines, consolidated survey and interview guide.

### Stage 1: Framework Development and Validation

**Figure 1:**
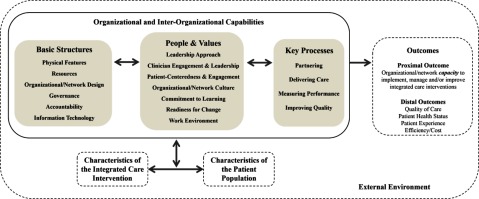
The Context and Capabilities for Integrating Care (CCIC) Framework [15].

We developed the Context and Capabilities for Integrating Care (CCIC) Framework (Figure 1) to collate and organize a comprehensive list of organizational factors that influence integrated care, and to capture the high-level mechanisms by which they influence integrated care initiatives [13]. Some studies of integrated care initiatives examine selected and limited organizational factors, but there is no comprehensive framework to guide research. The CCIC Framework was developed through a literature review of system-level integrated care strategies using 114 papers identified through a previous review [14]. We extracted organizational factors from included papers and grouped them together under preliminary categories.

The framework was revised and validated through semi-structured interviews with 53 managers and care providers engaged in the implementation and operation of 38 integrated care networks in Ontario, Canada. A combination of purposeful and snowball sampling was used to identify networks and participants. Interviews consisted of open-ended questions to identify important organizational factors followed by graphic elicitation to obtain views on the framework. We coded the transcripts deductively based on the CCIC Framework using NVivo software. Codes were also generated inductively for factors mentioned by participants and not already reflected in the CCIC Framework. The CCIC Framework consists of 18 organizational factors. Because the framework is specific to integrated care, certain elements of prior frameworks take on more importance and are more detailed (e.g., Governance, Information Technology, Clinician Engagement and Leadership, Patient-Centeredness and Engagement, Delivering Care, and Improving Quality). Detailed methods on how the framework was developed are reported in a separate publication [15].

Below we describe the development of the data collection instruments in the research toolkit. We aimed to capture in the toolkit all factors identified by the CCIC Framework with minimal respondent burden and to enable triangulation of results. We therefore began by developing document and website review procedures as they represent the least intrusive method of data collection, followed by the identification and consolidation of high quality survey instruments and the development of interview guides to address any remaining factors not covered by the previous methods.

### Stage 2: Development of Document/Website Review Guidelines

We developed a list of potential sources (e.g., organizational documents and websites), from which relevant information on organizational context may be extracted. Using the factors outlined in the CCIC Framework as a guide, we identified key information to look for and record during document and website review. The lists of sources and of key information were identified, expanded, and modified through group discussion among research team members. The guidelines outlined in the toolkit were informed by a seminal publication on the use of document analysis as a qualitative research method [16].

### Stage 3: Identification, Assessment, and Selection of Survey Instruments

We conducted a rigorous search for validated surveys that measure the constructs in the CCIC Framework. Independent searches were conducted for *each* construct in the framework using OVID Medline and Google Scholar. Key inclusion criteria were: (a) availability of a sufficiently detailed description of the development and/or use of the quantitative instrument (or scale) to enable assessment of content, (b) availability of a full copy of the instrument, and (c) previous use of the instrument in a healthcare setting. The database searches were supplemented by an expert consultation involving investigators with the Health System Performance Research Network (www.hsprn.ca) and other health services management experts known to members of the research team. A total of 40 experts responded and collectively identified 30 tools.

Identified instruments were assessed based on their psychometric properties, respondent burden, and applicability to integrated care efforts in terms of prior use and language (i.e., survey items are not specific to a particular care setting or type of professional). All three criteria were considered equally in the assessment of instruments. In instances where instruments covered similar content and were comparably strong across the above criteria, the authors discussed the merits of each instrument until consensus was reached on the most appropriate instrument. While some of the surveys could be applied to other initiatives beyond integrated care (e.g., Organizational Culture Inventory, Change Readiness Survey), others are specific to initiatives involving inter-professional and inter-organizational relationships and therefore might not be widely applicable (Partnership Self-Assessment Tool, Measure of Network Integration). Detailed methods and results on the review of survey instruments are reported in a separate publication [13].

### Stage 4: Development of Interview Resources

We developed a semi-structured interview guide to elicit views on which organizational factors have been most and least influential in shaping implementation and performance in a given integrated care initiative. We also developed a repository of interview questions that measure content areas not covered, or inadequately covered, by the survey instruments. The interview guide and repository were developed iteratively by the team through discussion and pilot testing.

### Stage 5: Pilot Testing

The consolidated survey instrument and the semi-structured interview guide were piloted with four managers and three care providers working in four integrated care networks in Ontario. The provider survey was piloted with two care providers, the manager survey was piloted with two managers, and the interview guide was piloted with two managers and one provider. The providers consisted of two nurse practitioners and a physician, while the managers consisted of a care coordinator, two directors of care/clinical services, and one senior executive.

Survey pilot participants filled out either a paper-based or online version of the survey and participated in a 30 minute debriefing interview to provide feedback. Interview pilot participants were engaged in a cognitive interviews [17] in which they were asked to consider the structure and content of the interview questions rather than answering questions outright.

Pilot testing of the consolidated surveys resulted in minor changes to wording in the introduction and instructions, and to one survey item. Overall, pilot test participants completed the survey in approximately 40 minutes and found the instructions and questions easy to understand and complete. Minor changes were also made to the wording of instructions in the interview guide, and to the questions themselves, to improve clarity and to improve the flow of questions.

## Results

The CCIC Framework consists of eighteen organizational factors in three categories: Basic Structures, People and Values, and Key Processes (Figure 1 and Table 1). These factors can be examined within organizations and across partnering organizations in a network.

**Table 1:** Context and Capabilities for Integrating Care (CCIC) Framework: Definitions and Examples [15].

Concept	Definition	Examples

***Basic Structures***		
Physical Features	Structural and geographic characteristics of the organization/practice and network	organization/practice size and age, urban or rural location, facilities, geographic proximity of network members
Resources	Availability of tangible and intangible assets for ongoing operations at the organization/practice and for network activities	staffing, funding, knowledge, time, project management support, administrative support, brand/reputation
Governance	How the board or steering committee is organized and its activities to direct, manage and monitor the affairs of the organization/practice and network	board/committee composition, types of sub-committees, frequency of meetings, types of decisions made (e.g., extent of centralized planning and standardization)
Accountability	The mechanisms in place to ensure that people and organizations meet formal expectations in the organization/practice and network	regulations enforced by an authority (e.g., government), formal agreements between organizations (e.g., data sharing), organizational mandates, professional scope of practice
Information Technology	The availability and ease of use of technology-based communication and information storage mechanisms in the organization/practice and across the network	shared electronic medical records, email communication, video conferencing, data access and mining, tele-healthcare
Organizational/Network Design	The arrangement of units and roles and how they interact to accomplish tasks in the organization/practice and network	organizational chart (hierarchy), types of departments/programs, job descriptions, communication and decision-making channels (e.g., are they centralized or decentralized? formal or informal?)
***People and Values***		
Leadership Approach	The methods and behaviours used by formal leaders in the organization/practice or network (i.e., individual leaders, leadership teams, or lead organizations)	personal vision for the organization/practice or network, strategies used to empower staff, leadership style and competencies
Clinician Engagement & Leadership	The formal and informal roles held by clinicians in the organization/practice and network, particularly physicians, that enable them to buy-in to and steer change, and influence others	active involvement of clinicians in planning, leading or supporting new initiatives (e.g., clinical champions or directors, networks led by primary care practices)
Organizational/Network Culture*	Widely shared values and habits in the organization/practice or network	perceptions regarding what is important and what is appropriate behavior
Focus on Patient-Centeredness & Engagement	Commitment to placing patients at the center of clinical, organizational and network decision-making	collection and use of patient feedback, consideration for patient needs and preferences, patient input and representation on committees as a standard practice, patient involvement in co-designing services
Commitment to Learning	The existence of a set of values and practices that support ongoing development of new knowledge and insights within the organization/practice and network	experimentation encouraged and rewarded, forums for meeting with and learning from other organizations and external experts, time and resources to reflect on past performance
Work Environment	How employees perceive and experience their job and their workplace in the organization/practice and network	opportunity for input, job satisfaction, burnout
Readiness for Change	The extent to which organizations and individuals are willing and able to implement change in the organization/practice and network	attitudes toward change and toward new or innovative ideas, extent of fit between current vision/strategy and the change
***Key Processes***		
Partnering	The development and management of formal and informal connections between different organizations/practices	sharing information, sharing staff, engaging in collaborative problem-solving, building a common understanding and vision, exchanging knowledge, implementing referral and discharge/transfer agreements
Delivering Care	The methods used by providers in caring for patients in the organization/practice and network	inter-professional teamwork and joint care planning, use of standardized decision support tools, medical model vs. holistic model of care, shared patient-provider decision-making
Measuring Performance	The systematic collection of data about how well the organization/practice and network is meeting its goals	shared performance measurement framework, regular measurement and reporting, data access and mining
Improving Quality	The use of practices and processes that continuously enhance patient care in the organization/practice and network	providing quality improvement (QI) training to staff, systematic use of QI methods (e.g., process mapping, control charts), application of best practices

*Capabilities such as Focus on Patient-Centeredness and Engagement, Commitment to Learning and Readiness for Change may manifest in the culture of the organization or network.

The document and website review procedures focus on publicly available information and readily accessible information from internal organizational documents. Examples of organizational data recommended for collection as part of the document review include: organization age and size (Physical Features of the Organization), staff mix and financial standing (Resources), organization affiliations and the degree of hierarchy and centralization (Organizational Design), and board composition and involvement in quality of care (Governance).

We identified 83 survey instruments meeting our inclusion criteria. Based on the assessment criteria and team discussion, six surveys were selected for inclusion (in whole or in part):

1. Partnership Self-Assessment Tool (PSAT) [18]
2. Measure of Network Integration [19]
3. Team Climate Inventory [20]
4. Change Readiness Survey [21]
5. Survey of Organizational Attributes for Primary Care [22,23]
6. Organizational Culture Inventory [24]

These six instruments, or select scales within the instruments, were consolidated to create the “Organizing for Integrated Care” survey, which consists of approximately 100 items. Two versions of the survey were created, aimed at managers and care providers respectively. Table 2 maps each instrument to the factors it measures in the CCIC Framework.

**Table 2:** Organizational Factors Measured by Selected Instruments/Items. ✓ denotes the main area covered by instrument, ⨯ denotes other areas directly covered by one or more items, ○ denotes areas indirectly addressed in the instrument.

Selected Instruments	CCIC Factors (12/18)	Resources	Organizational/Network Design	Leadership Approach	Clinician Engagement & Leadership	Organizational/Network Culture	Commitment to Learning	Work Climate	Readiness for Change	Delivering Care (Teamwork)	Improving Quality	Partnering	Measuring Performance

**Team Climate Inventory** [20] (14 items)							⨯	⨯	⨯	✓			
**Organizational Culture Inventory** [24] (12 items)			○	○		✓		○	○		○		○
**Change Readiness Survey** [21] – 3 scales only (15 items)									✓				
**Survey of Organizational Attributes in Primary Care (SOAPC)** [22,23] – 3 scales only (14 items)			⨯		⨯		○	✓	⨯		✓		⨯
**Measure of Network Integration** [19] – 2 scales only (9 items)						⨯					⨯	✓	⨯
**Partnership Self-Assessment Tool (PSAT)** [18,26] – 6 scales only (38 items)		⨯		⨯				○		○		✓	⨯

The interview guide consists of two sections. In the first section, there are three open-ended questions (and eight prompts) aimed at characterizing the integrated care initiative in terms of successes, challenges, and the nature of relationships. The second section uses the CCIC Framework and five questions to elicit rankings of the most and least important organizational factors from the participant’s perspective.

The interview question repository provides questions on organizational factors not well covered by the surveys, such as Information Technology, Leadership Approach, Clinician Engagement and Leadership, Patient-Centeredness and Engagement, Commitment to Learning, and Delivering Care. The repository also includes questions on topics that *are* measured by the surveys, such as Improving Quality, teamwork (within “Delivering Care”), and Measuring Performance. However, the interview questions target different aspects of these topics than their corresponding surveys items. This overlap allows for a rich analysis and triangulation of data (See Table 3 for an example). A total of 20 questions are provided, not including prompts, and are organized by respondent group (managers, clinical and administrative staff, and clinical staff). Together, the interview guide and repository provide the opportunity to understand and probe qualitatively what is measured quantitatively by the survey instruments.

**Table 3:** An Example of the Use of Mixed Methods to Triangulate Data on Clinician Engagement and Leadership.

Method	Content

Document and Website Review	1. Clinician leadership of key committees and initiatives (particularly for quality and safety).
	2. Clinician involvement on the board.
	Possible sources of information include: organizational website, annual reports, strategic plans, policies and procedures, terms of reference, improvement plans, job descriptions, meeting minutes and evaluation reports.
Survey Instrument	Participatory Decision-Making Scale of the Survey of Organizational Attributes for Primary Care (SOAPC) [22,23]
	1. This is a very hierarchical organization: The decisions are made at the top, with little input from those doing the work.
	2. This practice encourages staff input for making changes and improvements.
	3. This practice encourages nursing and clinical staff input for making changes and improvements.
	Responses are measured on a 5-point Likert scale from “strongly disagree” to “strongly agree”.
Interview Question Repository	How engaged and active are you and other clinical staff members in organizational issues?
	Prompts: – To what extent do you participate in decisions regarding the organization (such as quality improvement and strategic planning)? – What strategies do managers use to engage clinical staff in processes like strategic planning and organizational change? – In past periods of change, how has the organization supported and engaged clinical staff?

## Discussion

The research toolkit was designed to yield standardized comparable data on organizational and inter-organizational factors that influence the implementation and success of integrated care initiatives. Below we offer guidance and considerations for the optimal use of the toolkit.

### Intended Users

The toolkit may be used by both researchers and managers interested in understanding the influence of organizational context on integrated care initiatives. When used in research, the toolkit can help characterize and compare organizational context across multiple cases and enable comparison of results across studies. This knowledge can help explain why some integrated care initiatives succeed and some fail, and can be used to generalize findings and best practices across integrated care settings.

We also encourage the use of the toolkit by leaders and managers engaged in planning, implementing or evaluating integrated care initiatives. Application of the toolkit can provide information and support to managers in identifying appropriate partner organizations and change management strategies.

### Intended Settings and Respondents

The toolkit is intended for application within and across the organizations involved in a given integrated care initiative. This may include primary care practices, community care organizations, hospitals, long-term care facilities, allied health organizations, and social services agencies, among others. In order to examine the organizational and inter-organizational factors within and across these organizations, appropriate respondents include senior management, middle management, and clinical staff. Administrative staff may also be included. When applying the toolkit to several different organizations, terms in the toolkit such as “organization” and “practice” can be used interchangeably depending on the participant.

Sampling approaches will vary based on the network, organization or team size. In a small organization, or in a small integrated care network or team, no sampling will be necessary. Rather, all involved staff may be invited to participate in the surveys, and potentially the interviews depending on available resources. In larger teams, organizations, and networks, stratified random sampling or a combination of purposive and snowball sampling may be used. Together, participants should represent all hierarchical levels and both administrative and clinical roles.

### Measurement Timing

The toolkit may be applied at various points in the life cycle of an integrated care initiative, including planning and design, during implementation, or post-implementation. Pre-implementation, the toolkit may be used to determine readiness to integrate, to select partners with overlapping or complementary contextual characteristics, or to predict and address potential problems. During implementation, the toolkit may be used to shape change management and conflict management strategies in real-time. Finally, post-implementation, the toolkit may be used as part of a broader evaluation of the success and sustainability of integrated care initiatives.

### Boundary between the Organizational Context and the Intervention

Challenges may emerge around differentiating between the organizational context and the characteristics of the integrated care initiative itself. Integrated care initiatives often involve the implementation of new clinical processes, models of teamwork, and inter-organizational collaboration – all factors that the toolkit measures. Therefore, application of the toolkit will produce data with relevance to understanding and assessing *both* the integrated care initiative and its organizational/network context.

### Modification of the Toolkit

The toolkit is composed of various sections, including multiple survey scales, document/website review procedures and semi-structured interview resources. Though many of these tools can be applied independently, we strongly recommend using the toolkit as a whole for two reasons. First, the various parts of the toolkit were created to both complement one another, and to overlap where appropriate. Therefore, any major modifications to the toolkit may result in missing information on one or more factors in the CCIC Framework. Second, application of the entire contents of the toolkit ensure complete and comparable data collection across settings and studies. Furthermore, development of the toolkit involved consideration for participant burden due to lengthy surveys and interviews. The length and complexity of the tools have thus been balanced with the need for comprehensive coverage of the factors identified in the CCIC Framework, resulting in some instruments not being used in their entirety. While scales were kept intact to preserve internal reliability, future research should involve validity testing of the consolidated survey in the toolkit.

Some modifications to the toolkit may be appropriate depending on the research question, user needs and the setting. However, any modification should take into account potential issues with the validity of survey scales and reduction in the comparability of the data across diverse settings and studies. We offer additional guidelines below to inform potential modifications.

If using the full recommended survey is not feasible, we suggest the use of the interview guide to inform which survey scales to use. The interview guide elicits participant opinions on the most important factors in the CCIC Framework. Based on the results of the interview, a tailored, context-specific set of survey scales may be used to collect data on the factors identified as most important.

The interview guide consists of two sections. The first section asks open-ended questions, while the second section involves presenting the participant with a copy of the CCIC Framework and eliciting information on the relative importance of the factors in the Framework based on their experiences. The first open-ended section may be removed if there are time constraints, but its use is recommended as a way to obtain unbiased responses from participants before showing them the Framework. Furthermore, the risk of over-simplifying contextual factors using surveys is tempered by the inclusion of a semi-structured interview guide. The open-ended interview questions enable researchers and managers to engage with and capture the complexity of integrated care initiatives and the organizational contexts in which they are implemented.

For researchers or managers interested in probing further on factors not measured by the survey, the toolkit provides an Interview Question Repository with additional questions that can be inserted into the interview guide. The repository contains questions that either drill down further into concepts explored in the survey or cover areas from the CCIC framework that are not measured by the survey. The use of these questions is optional.

The research toolkit we developed complements and extends Project INTEGRATE, a European initiative consisting of a common set of tools for evaluating and comparing integrated care initiatives [25]. Their data collection tools include a case study report template that incorporates guidelines for retrospective process and data analysis as well as semi-structured interviews with stakeholders. Their methods focus primarily on describing integrated care initiatives and their implementation. A section on the context for integrated care is also included in which five broad, open-ended questions are provided to aid in the identification of key enablers and barriers. While there is some overlap in the content of our respective interview guides, our toolkit focuses on the (inter-)organizational context and includes explicit document review guidelines as well as survey instruments. As such, our toolkit may be implemented alongside the methods proposed by Project INTEGRATE.

### Limitations

The toolkit is currently being used in one evaluation of a province-wide integrated care initiative in Ontario, Canada (Health Links) and in one international study of integrated care (the Integrating Care for Older Adults with Complex Health Needs (ICOACH) program, involving Ontario, Quebec and New Zealand). However, further validation of both the CCIC Framework and the toolkit in other healthcare systems is warranted. The relative value of the toolkit to researchers and practitioners also needs to be examined. Furthermore, the potential to compare organizational context across settings and studies is dependent on the consistent and timely gathering of data across comparable sites. Finally, the toolkit does not explicitly measure the *impact* of organizational context on integrated care, but rather describes and characterizes organizational context. Future research should leverage the toolkit to examine relationships between organizational context factors and integrated care processes and outcomes. Examining how organizational context interacts with individual characteristics and environmental factors to shape integrated care delivery is also a fruitful direction for future research.

## Conclusion

The research toolkit provides a standardized approach for conducting case studies on the organizational context for integrated care delivery. The toolkit can be used to characterize and compare organizational factors across multiple cases and enable comparison of results across multiple studies. The use of standardized quantitative and qualitative instruments facilitates longitudinal data collection to help inform our understanding of the dynamic interactions and evolution of organizational context factors. This information can enhance our understanding of the influence of these factors, support the transfer of best practices, and help explain why some integrated care initiatives succeed and some fail. This information may also inform implementation and change management strategies for future integrated care efforts by prioritizing key organizational factors that require modification or strengthening to enhance the probability of success. We invite researchers and practitioners to contact us for access and permission to use the toolkit.
